# AIMNet2: a neural network potential to meet your neutral, charged, organic, and elemental-organic needs†

**DOI:** 10.1039/d4sc08572h

**Published:** 2025-04-29

**Authors:** Dylan M. Anstine, Roman Zubatyuk, Olexandr Isayev

**Affiliations:** a Department of Chemistry, Mellon College of Science, Carnegie Mellon University Pittsburgh Pennsylvania 15213 USA olexandr@olexandrisayev.com

## Abstract

Machine learned interatomic potentials (MLIPs) are reshaping computational chemistry practices because of their ability to drastically exceed the accuracy-length/time scale tradeoff. Despite this attraction, the benefits of such efficiency are only impactful when an MLIP uniquely enables insight into a target system or is broadly transferable outside of the training dataset. In this work, we present the 2^nd^ generation of our atoms-in-molecules neural network potential (AIMNet2), which is applicable to species composed of up to 14 chemical elements in both neutral and charged states, making it a valuable method for modeling the majority of non-metallic compounds. Using an exhaustive dataset of 2 × 10^7^ hybrid DFT level of theory quantum chemical calculations, AIMNet2 combines ML-parameterized short-range and physics-based long-range terms to attain generalizability that reaches from simple organics to diverse molecules with “exotic” element-organic bonding. We show that AIMNet2 outperforms semi-empirical GFN2-xTB and is on par with reference density functional theory for interaction energy contributions, conformer search tasks, torsion rotation profiles, and molecular-to-macromolecular geometry optimization. Overall, the demonstrated chemical coverage and computational efficiency of AIMNet2 is a significant step toward providing access to MLIPs that avoid the crucial limitation of curating additional quantum chemical data and retraining with each new application.

## Abstract 2

Machine learned interatomic potentials (MLIPs) are reshaping computational chemistry practices because of their ability to drastically exceed the accuracy-length/time scale tradeoff.

## Graphical abstract

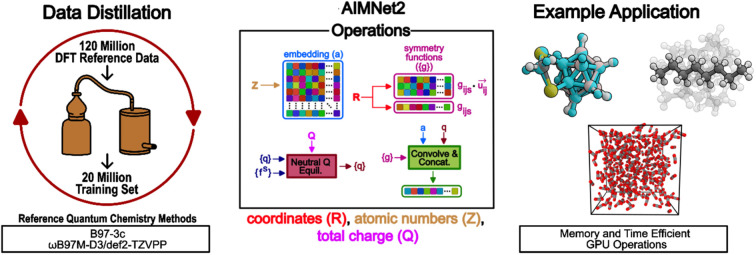

## Introduction

The accessibility of quantum mechanical (QM) calculations and the continuous improvement of data-driven techniques, such as machine learning, have unlocked chemistry research directions that would be otherwise too expensive or impractical to pursue.^1–3^ Machine learned interatomic potentials (MLIPs)^4–6^—which are models that aim to reproduce QM potential energy surfaces given sufficient training data—have a notable presence in this emerging style of chemical research. One of the main attractions of these models is that quantum chemical calculation workloads that require hours or days can be approximated within seconds. Using MLIPs, it is now possible to examine large batches of molecular systems or materials consisting of >10^5^ atoms with minimal sacrifices compared to QM accuracy using relatively modest computational resources, if pretrained models are made available. Computational chemistry research has accelerated to a point where evaluating millions of systems is trending toward becoming a routine step in the design-of-experiments, albeit with access to the proper accelerated computing hardware. As a result, MLIPs exist as promising tools for addressing diverse challenges faced across the chemical sciences,^7–9^ especially if they are robust enough to be coupled with high-throughput experimentation, autonomous synthesis platforms, and robotic chemistry laboratories.^10–13^

Avoiding the cost of QM calculations is a primary MLIP benefit; however, most reported models are either specific to one system or a small number of compounds. This slows the ability of MLIP-driven simulations to address chemical challenges, particularly when QM data is not available and needs to be generated. The time required to curate a dataset, train an MLIP, and properly validate its chemical space coverage can significantly offset the low computational cost of applying the model. An alternative is to collect a large amount of training data with broad chemical space coverage and train a general MLIP, ideally with a workflow that minimizes unnecessary QM calculations and maximizes the contribution each system has for refining a model.^14–16^

With this motivation, there is a need to develop MLIPs that are transferable to a wide range of compounds with diverse chemical compositions and charge spin states. The accurate neural network engine for molecular energies (ANI)^17,18^ family of MLIPs were some of the earliest models to achieve reliable predictions for millions of molecular systems composed of H, C, N, O, F, Cl, and S.^19^ The ANI MLIPs are effective for cases where physical and chemical characteristics can reasonably be approximated using only short-range truncated chemical environments; however, different model architectures are required for systems with many elements, non-local behavior, open-shells, and charged species. Recent model developments have overcome the poor scaling with respect to the number of parametrized chemical elements, provided mechanisms to incorporate contributions from long-range interactions,^20–23^ and introduced methods for considering spin states.^24–26^ Herein, we report an advancement to the atom-in-molecules neural network potential model suite, AIMNet2, which expands our previous model to include 14 chemical elements and long-range electrostatic and dispersion interactions for compounds with varied charges and valency. In addition to making these pretrained models available, we also provide access to the AIMNet2 architecture, allowing the computational chemistry community interested in developing MLIPs to train their own models and fully utilize the efficiency and scalability for targeted applications.

## Results and discussions

### Model design

**Fig. 1:**
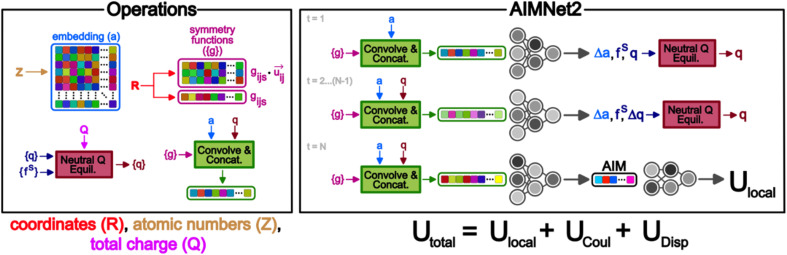
Operations and unrolled message passing workflow of the AIMNet2 architecture. Atomic coordinates (*R*), atomic numbers (*Z*), and net charge of the system (*Q*) are model inputs. AIMNet2 uses a message passing approach, where atomic feature vectors (*v*(*)) are calculated *via* a convolution and concatenation of atomic and geometric descriptors. The local configurational energy is obtained using the atoms-in-molecule vector (AIM), which is summed with dispersion and electrostatic contributions in the calculation of the total energy.

A schematic overview of the key components of the AIMNet2 architecture is shown in Fig. 1. AIMNet2 calculates the total energy of a chemical system according to

1 *U*_Total_ = *U*_Local_ + *U*_Disp_ + *U*_Coul_

where *U*_Local_, *U*_Disp_, and *U*_Coul_ refer to the local configurational interaction energy, explicit dispersion correction, and electrostatics between atom-centered partial point charges, respectively. Similar to the previous version of AIMNet,^27^ multi-task predictions can be constructed on-top of the learned representation, *i.e.*, the so-called AIM vector, but we chose to omit them for clarity. However, this feature supports the flexibility of AIMNet2 to be applied to diverse molecular and material systems because the functional form can be readily tailored to meet the demands of the modeling task by including additional output heads. We include explicit dispersion interactions using a PyTorch^28^ implementation of the DFT-D3 correction model from Grimme and coworkers.^29,30^ All source code and pretrained models used in this work are provided in the open-source repository https://github.com/isayevlab/aimnetcentral on GitHub.

In AIMNet2, the AIM layer is a learned atomic representation that is determined using a message-passing architecture. First, the interatomic distances are expanded into a set of radial symmetry basis functions of the form:2
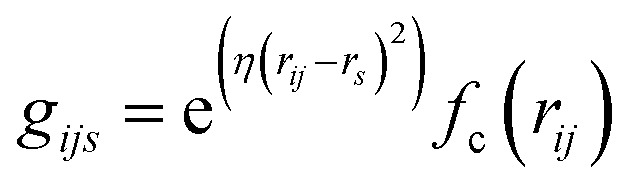
where the local atomic environment of atom *i* is described as a collection of Gaussian functions with a set of center positions *r*_*s*_ and widths *η*. The subscript dimension *s* defines the number of Gaussian functions composing the basis (16). The symmetry functions are damped with a cosine cutoff function (*f*_c_) that smoothly reduces these descriptors to 0 at the local distance cutoff of 5.0 Å. It should be noted that this cutoff is used only in evaluation of *U*_Local_, while long-range interactions, such as *U*_Coul_ and *U*_Disp_, are calculated for the entire system, or with a suitable cutoff, *e.g.* 15 Å. The strategy of augmenting short-range interactions with long-range contributions is one of several approaches to overcome the nearsightedness of MLIPs, where methodological trade-offs have recently been discussed in detail.^23^ Regardless, the atomic environment vectors are combined with atomic embeddings (eqn (3)–(5)) to provide a feature vector representation that is rich in chemical details.

3 *a*_*ds*_(*z*) ∈ *R*^*ds*^

4
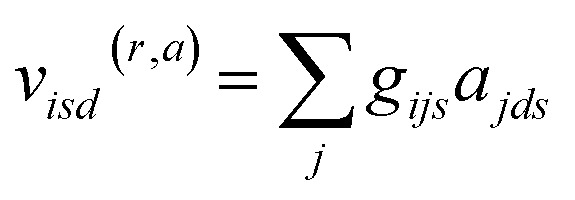
5
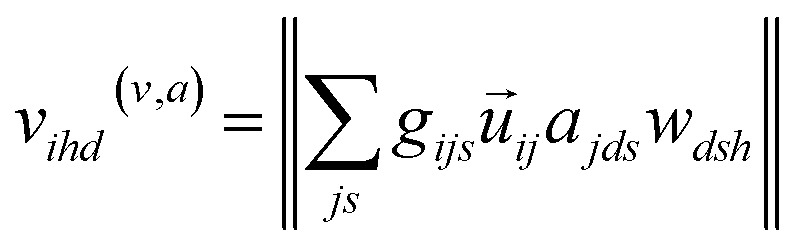


Atomic embeddings (*a*) are defined using a 16 × 16-matrix (*d*,*s*) that initially depends on each atom's atomic number (*z*), where *d* is a hyperparameter controlling the embedding size. The design of this 2D-embedding was motivated by a desire to enhance AIMNet2's flexibility by introducing a message-passing convolution that depends on which radials shells dominate the composition of *g*_*ijs*_. With each message pass, the atomic embedding is updated to provide a refined description of the chemical environment of neighboring atoms, thus, obfuscating the need for multiple element-specific networks, which are required, for instance, in MLIP models such as ANI.^17,18^ This flexibility provides the AIMNet2 architecture an ability to efficiently generalize to arbitrary number of chemical elements without species-specific networks. During the first message passing iteration, the atomic feature vectors are constructed *via* a concatenation of so-called ‘scalar’ (*v*_*isd*_^(*r*,*a*)^) and ‘vector’ (*v*_*ihd*_^(*r*,*a*)^) embedding components, which collect information of the atomic environment using harmonics with angular momentum *l* = 0 and *l* = 1. The *v*_*ihd*_^(*r*,*a*)^ calculation is similar to that of *v*_*isd*_^(*r*,*a*)^; however, a combination of the embedding features is carried out using linear transformation with the weight matrix, *w*_*dsh*_, before performing a vector-norm of the resultant matrix multiplication sum. A set of initial atom-centered partial point charges (*q*) are predicted during the first message pass. In subsequent iterations, the input description of each atom is expanded to include charge components. Partial charges undergo a similar convolution to that described in eqn (4) and (5); however, *a*_*ds*_ is replaced with each atom's partial point charge. Thus, the atomic feature vectors after the first message pass are modified to be a concatenation of *v*_*isd*_^(*r*,*a*)^, *v*_*is*_^(*r*,*q*)^, *v*_*ihd*_^(*r*,*a*)^, and *v*_*ihd*_^(*r*,*q*)^.

It is worth highlighting that other models have been reported that include electronic structure information, *e.g.*, partial charges, as a component in their input representation. As an example, one could use partial charges from charge equilibration procedures (QEq),^31^ as is done in the 4GNNP model of Ko *et al.*,^21^ which requires defining environment-dependent electronegativities and solving a system of linear equations either iteratively or through matrix inversion. In contrast, AIMNet2 infers partial charges from the feature vector representation and iteratively refines them as part of the message passing procedure. Every partial charge update is followed by an application of Neural Charge Equilibration (NQE), which is a methodology adapted from the work of Zubatyuk *et al.* for simulating open-shell or ionic species with AIMNet-NSE:^25^6
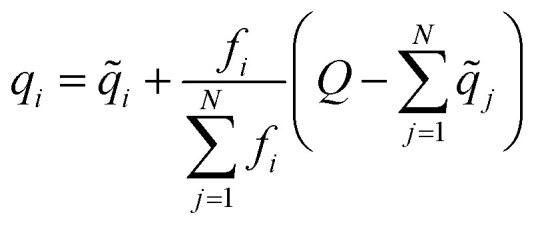
where *q̃* and *q* are partial atomic charges before and after equilibration, *f* is a non-negative atomic weigh factor which is predicted alongside the partial charges, and *Q* is total molecular charge. This normalization procedure re-distributes any surplus or deficit partial charge along the atoms of the molecule according to predicted weights.

The final message passing iteration yields the AIM representation, which serves as the input for a feed-forward neural network block that ultimately is used to infer *U*_Local_.

### Data distillation

A major challenge to training an MLIP that covers wide ranges of chemical space is that the reference dataset used during training can quickly grow to an impractical scale. As a result, it is necessary to carry out data curation and model training practices that limit dataset redundancy and maximize the value that each data point will contribute to refining the MLIP model. The overall aim is to achieve a manageable collection of informative quantum chemical data that can be used to train an AIMNet2 model that displays similar accuracy to a model that is laboriously trained on the full set of labeled data. In this report, we compact our dataset by implementing a strategy that we refer to as data distillation.

The process of data distillation involves sequentially growing a training set that is a subset of the master set of all the accumulated quantum chemical calculation results, *i.e.*, ∼120 million samples (molecular systems) labeled with low-fidelity B97-3c^32^ DFT method. Specifically, we began by randomly selecting 1 × 10^5^ reference data and trained an initial AIMNet2 potential. Following training, we performed inference on the master set, with molecules sorted from smallest to largest, until we found an additional 1 × 10^5^ reference data that are predicted above a threshold of 3× the current training error. Candidate structures from the master set were evaluated using both force and energy criteria, where samples falling above either error threshold, defined using the most recent training run, were selected. These structures are added to the training set, and training continues starting from the previous model weights. This process repeats until the final AIMNet2 model can accurately describe the entire master set, which occurred for our pretrained AIMNet2 models when we reached ∼2 × 10^7^ reference data points. We then retrained the final ensemble AIMNet2 models (4 members) from scratch.

**Fig. 2:**
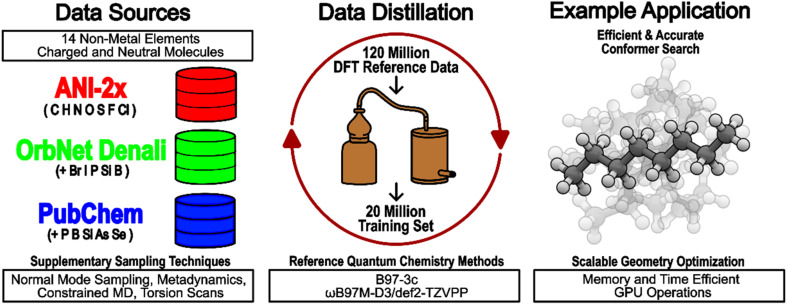
Overview of AIMNet2 model development and application usage. Diverse sampling techniques were used to curate a dataset of 120 million chemical systems that were labeled with B97-3c DFT. Following data distillation, the remaining 20 million systems were labeled with ωB97M-D3/def2-TZVPP and used to train the application ready AIMNet2 models.

An overview of the preparation and use of our pretrained AIMNet2 models is presented in Fig. 2. We used ChEMBL^33^ and PubChem^34^ as key sources of the molecular structures. We performed non-equilibrium conformational sampling with molecular dynamics and metadynamics using GFN2-xTB^35^ and torsional scans with preliminary models. Additional structures were added from ANI-2x^18^ and OrbNet^36^ datasets. Altogether, this formed the master set of ∼1.2 × 10^8^ molecular conformers for data distillation. The entire pool of structures was initially labeled with computationally efficient B97-3c^32^ calculations. After reducing the master dataset to ∼2 × 10^7^ samples, all structures were computed with more expensive and accurate ωB97M-D3/def2-TZVPP.^37^ All reference DFT data were used in training without any dispersion correction. In the final models, 2-body DFT-D3 [https://doi.org/10.1002/jcc.21759] dispersion correction was added with damping function parameters for B97-3c and ωB97M. respectively. Additional details and statistics regarding the dataset can be found in the Methods section and ESI.†

### Case study of uncommon bonding

In this section, we report two test cases using our pretrained AIMNet2 models to demonstrate transferability. In the first case we consider the ability of the AIMNet2 models for reproducing experimentally observed geometries of molecules with unusual bonding. For the second test case, we assess performance in conformer search tasks for species with an extended set of chemical elements with verified experimental crystal structures. The aim of the first benchmark is to highlight that the potential energy surface learned by the pretrained models can be used to accurately identify molecular geometry minima for organic and element-organic structures, particularly those with diverse covalent bonding. To emphasize this robustness, we selected 113 molecular structures that have rare bonding patterns from a larger extracted set from the Cambridge Structural Database (CSD).^38^ For details on the criteria and procedure used to down select these structures from an initial set of ∼2.5 × 10^5^ diverse compounds, see the Methods section and ESI Note 1.† While AIMNet2 models were trained on samples broadly containing the covalent bonding possible in our element set, these testing molecules show rare chemical bonding patterns and therefore are borderline and challenging cases for an atomistic potential.

**Fig. 3:**
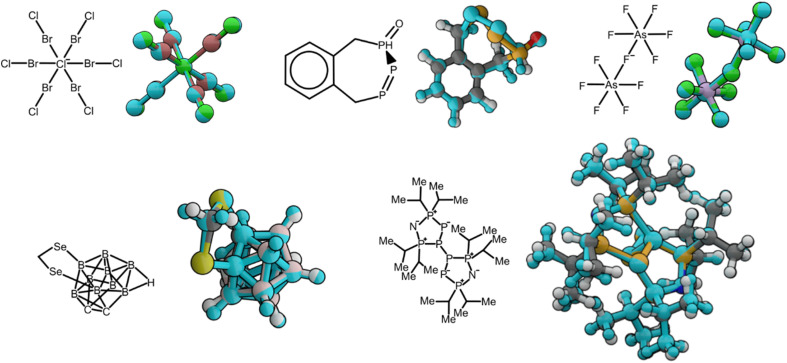
Alignment of molecular geometries optimized with AIMNet2 in the gas phase compared to conformers extracted from the experimental crystal structure. 2D molecular sketches are depicted along their corresponding 3D geometries. Experimental conformers are colored with the SMARTS color scheme, and AIMNet2 optimized structures are colored in light blue regardless of atom type.

For each structure, geometries were optimized with the pretrained AIMNet2 models in the gas phase and compared to a ground truth conformer extracted from experimentally resolved crystal structures. For these 113 selected molecules (see the ESI† for geometries and reference codes) our models displayed an average root-mean-squared deviation (RMSD) of 0.38 Å. Six examples from the 113 total cases evaluated are presented in Fig. 3. Considering some discrepancy is expected when comparing gas phase calculations and crystal structure geometries, the low RMSD value shows our model is robust even for fringe cases like a six-coordinated Cl ion or a selenium-doped boron cluster. In addition to assessing the AIMNet2 models trained on the results of ωB97M-D3 calculations, we compared with GFN2-xTB and AIMNet2 trained to B97-3c reference data. Geometry optimization was carried out with reasonably tight convergence criteria (*f*_max_ < 5 × 10^−3^ eV Å^−1^) starting from experimental geometry, which was followed by computing the RMSD of heavy atom positions between the experimental and optimized geometries (see ESI Table 2 and Fig. 1†). Both AIMNet2 models were observed to yield lower RMSDs (0.32 and 0.35 Å) compared to those from semi-empirical GFN2-xTB (0.37 A). We also examined the lengths of bonds containing non-hydrogen atoms and at least one species from our so-called “extended element” set (B, Si, P, As, Se, Br, I) to provide further insight into the ability of AIMNet2 to accurately describe diverse chemical geometries. The mean absolute deviation in these bond lengths from our 113 molecules is 2.4% and 2.1% for AIMNet2-B973c and AIMNet2-ωB97M-D3, respectively, indicating that, despite their uncommon nature, AIMNet2 captures extended element covalent bonding within an accuracy of a few picometers. It should be noted that two structures, [As_3_Br_12_]^3−^ and [As_3_I_12_]^3−^ (Refcodes VUFRIX and GEHVIY), decomposed into two fragments during optimization with GFN2-xTB, and the latter also with AIMNet2-ωB97M-D3. These entries were excluded from the RMSD statistics. Moreover, four structures failed to converge during the self-consistent charge procedure of GFN2-xTB. Regardless, these results show that AIMNet2 can reliably reproduce molecular geometries even in unusual, arguably exotic, bonding situations.

In the second step of our benchmark study, we measured the performance of pretrained AIMNet2 models in a conformational search task (see ESI Note 2†). We define success in this task as the ability to identify conformers that agree with those resolved experimentally by starting from a consistent pool of structures that were generated from molecular graphs without bias toward the ground truths, *i.e.*, the geometries extracted from the CSD. For an interatomic potential to be used in conformer search, it must describe interactions between particles in near and off-equilibrium molecular geometries accurately, thus, success in this benchmark supports the broad chemical space coverage of AIMNet2. Beginning with the same subset of ∼2.5 × 10^5^ extracted molecules, we selected a chemically varied set of 676 molecules that have 10–40 non-H atoms and 1–3 rotatable bonds. From each molecule's SMILES representation, an initial pool of molecular structures was produced using torsion driving with OpenEye Omega's Dense conformer ensemble generator. On average, 86 distinct conformers were generated for each molecule. After optimizing all conformers within the ensemble, we selected only those within 6 kcal mol^−1^ from the lowest energy conformer, which is a typical energy cutoff used in a conformation search task. Then within the pool of low-energy conformers, we searched for the conformation that is the closest to the experimental structure and recorded its RMSD and relative energy within the ensemble.

**Fig. 4:**
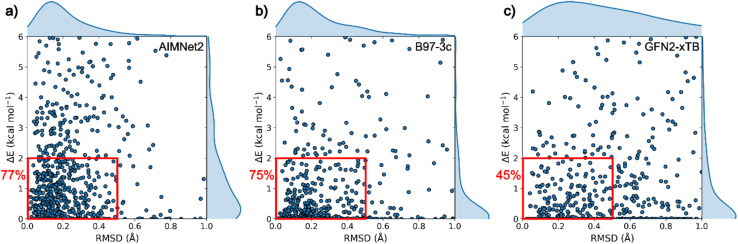
Success rate (red squares) of matching experimental geometries in a conformer search task of extended element structures. Success can be judged by the criteria of being low-energy (<2.0 kcal mol^−1^) and having low root-mean-squared-deviation (RMSD < 0.5 Å). Each data point represents the single closest match to the experimental structure extracted from CSD for each of the 676 targets. A consistent set of structures is evaluated for (a) AIMNet2, (b) B97-3c, and (c) GFN2-xTB.

In Fig. 4, we compare the success rate in locating approximate experimental geometries within the set of low-energy conformers in the optimized pool of structures for low-cost DFT, semi-empirical GFN2-xTB, and AIMNet2. We define a broad metric of success using two criteria: (1) the number of structures that have a low (<0.5 Å) RMSD to the ground truth and (2) the lowest RMSD structure also displaying low-relatively energy (<2.0 kcal mol^−1^) in the optimized pool. In other words, these criteria (displayed as red boxes in Fig. 4) reflect the likelihood of finding a high-quality molecular geometry if one were to conduct conformer search without knowing the ground truth. It is worth acknowledging that the bounds of this success window are somewhat arbitrary, and they can be tailored for the application or molecule(s) of interest. To limit ambiguity in our definition of success, the distribution of closest matches for each method are provided along the external bounds of Fig. 4. The pretrained AIMNet2 models display the lowest average RMSD and most compact distribution for identifying the experimental geometry among the three methods. Interestingly, GFN2-xTB optimizations result in better, on average, energy predictions than AIMNet2; however, this should be balanced against the significantly larger breadth of the distribution in the geometric comparison. In other words, many of these low energy predictions experience large geometric deviations that can hinder their practical use. Conformer search using DFT (B97-3c) optimizations can be regarded as a reliable, albeit more computationally demanding, measure of the typical success rate for this benchmark. Since performing optimization with the hybrid DFT method is computationally demanding, the DFT results are reported using B97-3c^32^ which serves as a reasonable reference point. Overall, this method^32^ identifies conformer geometries that are close to experimentally observed structures in 83% of the cases (see ESI†). It should be noted that this reflects not only the accuracy of the method but also the quality of conformational ensembles produced by OpenEye Omega, which is out of scope of our benchmark. The small percentage of geometries that are outside the RMSD window should not necessarily be labeled as a failure of the DFT or AIMNet2 potential energy surface representations, but instead they reflect a population of higher deviation minima. Considering both energy and geometry criteria, B97-3c conformer search was found to achieve success in 75% of the 676 cases (see Fig. 4b). The success rate for neutral molecules is observed to be ∼15% higher compared to charged ones. GFN-FF^39^ (see ESI†) and GFN2-xTB methods displayed noticeably lower success rates, especially for conformer geometry (see ESI Table 3†), with values of 42.1% and 45.2%, respectively. In contrast, AIMNet2 models trained on ωB97M DFT data achieved a 77% success rate and is within 2% of direct B97-3c calculations for both criteria.

### General interaction energy benchmarks

To evaluate the performance of AIMNet2, we examined two of the most extensive and chemically diverse validation data sets available for discerning accuracy in quantum chemical calculations, namely GMTKN55 (ref. 40) (General Main-group Thermochemistry, Kinetics, and Noncovalent interactions) and NCI Atlas (Non-Covalent Interactions Atlas).^41–44^ Both benchmarks are designed for targeted assement of the accuracy of electronic structure calculation methods for describing various chemical behavior. The GMTKN55 (ref. 40) validation set of Goerigk, Grimme, and co-workers is divided into 55 sub-datasets, where each focuses on specific phenomena underpinning molecular properties. In particular, there are seven datasets that address reaction barrier heights, 18 datasets dedicated to basis properties and smaller molecular systems—where nine of these primarily investigate noncovalent intramolecular interactions, 12 datasets consist of diverse intermolecular interactions, and the remaining nine are concentrated on reaction energies and isomerization energies for larger systems. The NCI Atlas is a curated collection of interaction energies and dissociation curves for complexes where intermolecular interactions are dominated by contributions such as London dispersion, sigma–hole interactions, and hydrogen bonding in charged and neutral molecules (including extended species: B, S, Se, P, halogens). Compared to earlier datasets like S66,^45^ the NCI Atlas datasets are larger, more accurate, and they also offer additional advantages such as a systematic construction, increased diversity of the model systems, and high-quality molecular geometries, to name a few.^44^

Typically, when evaluating the performance of QM methods using the GMTKN55 benchmark, results are reported using aggregated scores known as WTMAD1 or WTMAD2. These scores are derived by weighing the mean absolute deviation of the calculated results against the reference values. The distinction between WTMAD1 and WTMAD2 lies in the relative weighting assigned to the different subsets within GMTKN55.

**Fig. 5:**
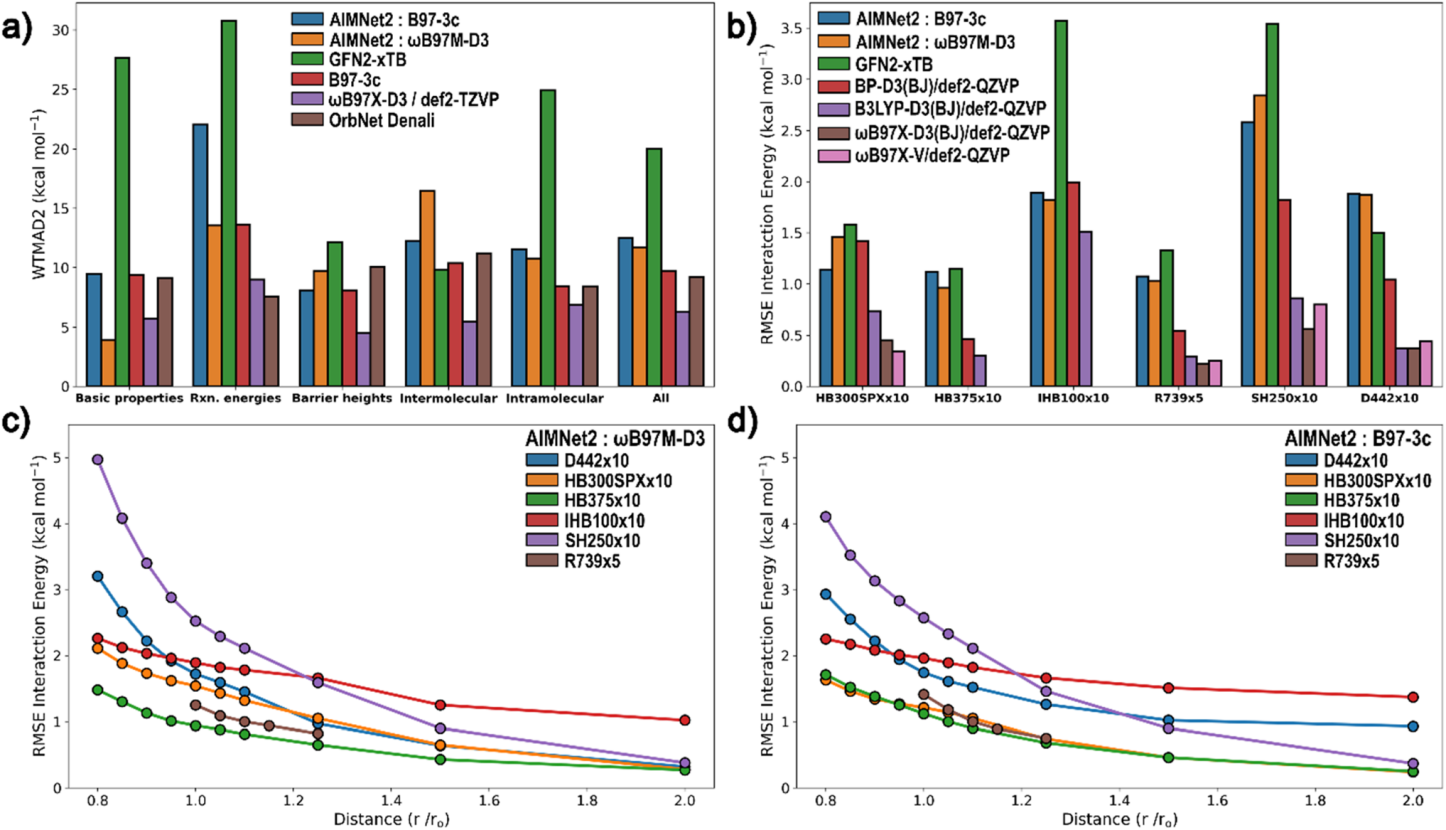
Performance of AIMNet2 models, GFN2-xTB and DFT methods on the (a) GMNTK55 benchmark and (b) the Non-Covalent Interaction (NCI) Atlas benchmark. Performance as a function of separation distance is reported for AIMNet2 models trained to ωB97M-D3 (c) and B97-3c (d) for the NCI Atlas benchmark. HB300SPX×10 – hydrogen bonding extended to S, P and halogens; HB375×10 – hydrogen bonding in organic molecules; IHB100×10 – ionic hydrogen bonds in organic molecules; R739×5 – repulsive contacts in an extended chemical space; SH250×10 – sigma–hole interactions; D442×10 – London dispersion in an extended chemical space.

Consistent with the OrbNet Denali report^46^ and to enable a fair comparison between models with varying coverage of elements, charge and spin states, we calculated WTMAD scores over the GMTKN55 subsets that are supported for each model and set the weight to 0 for the mean absolute deviation (MAD) for unsupported subsets. Fig. 5a lists WTMAD2 scores of AIMNet2 models trained to two DFT references B97-3c and ωB97M-D3/def2-TZVPP levels. Both models achieve substantial accuracy improvements compared to low-cost semi-empirical GFN2-xTB and are approximately equal to the proprietary OrbNet Denali model.

The only subset of the GMTKN55 dataset where the accuracy of AIMNet2 models does not outperform GFN2-xTB is for intermolecular interactions, which provides motivation to pursue additional detailed assessment for various types of noncovalent interactions to better understand the performance of AIMNet2. We further investigated the intermolecular interaction performance using NCI Atlas, where noncovalent interactions are partitioned into different types in a defined chemical space. For this benchmark, the AIMNet2 models significantly outperform for the subsets of ionic hydrogen bonds (IHB100×10) and sigma–hole interactions (SH250×10), whereas GFN2-xTB displays higher accuracy for the subset dispersion-bound molecular complexes (D442×10) by ∼0.3 kcal mol^−1^. These results are well aligned with the intermolecular interaction findings for GMTKN-55, whose subset consists of many dispersion bound systems such as π-stacked and nonpolar complexes. The overall performance of AIMNet2 is, on average, 1–2 kcal mol^−1^ RMSE for the various subsets of NCI Atlas (See Fig. 5b), which demonstrates notable accuracy improvements for electrostatics and directional intermolecular bonding. This is nearly twice as large as the typical errors reported for DFT methods; however, it represents a 25–50% improvement in accuracy for ionic hydrogen bonds and sigma–hole interactions over GFN2-xTB. It is important to place the prediction accuracy of interaction energies in the context of separation distance. In Fig. 5c and d, it is shown that the aggregate RMSE metrics are mainly dominated by differences occurring at separations less than the equilibrium or reference spacing, depending on the subset. The most significant difference is found for short-range sigma–hole interactions, which we regard as challenging physicochemical behavior to accurately predict for an atom-centered point charge model, especially one relying on local environment descriptors. A similar plot for GFN2-xTB accuracy as a function of distance is provided in Fig. S5.† The same trends observed for our pretrained AIMNet2 models are displayed, albeit with GFN2-xTB producing significantly larger errors for most distances and subsets.

It is worth commenting on the robustness of the pretrained AIMNet2 model errors with respect to predicting interaction energies of systems with varied total molecular charge (see ESI Fig. 4†). By comparing different subsets of our training data, including neutral (*Q* = 0), charged (|*Q*| ≤ 2), and strongly charged (|*Q*| from 3 to 9), we observe a consistently low ∼1.5 kcal mol^−1^ RMSE. In other words, there is not a clear discernible bias of the model error as a function of the total molecular charge.

To enable a comparison with models trained to a common set of chemical elements (CHNOSFCl) we also benchmarked the AIMNet2 model on the TorsionNet500 (ref. 47) dataset of torsion energy profiles for typical drug-like fragments. Following the outline of the original TorsionNet500 report, we compared several different metrics of accuracy (See Table 1). The AIMNet2 model shows a substantial improvement from the ANI-2x model, resulting in 3-5x error reduction and improvement in coverage while maintaining effectively the same computational performance. Torsion profiles calculated using OrbNet Denali and B97-3c are also considered, where the AIMNet2 model displays performance that is consistent with B97-3c and ∼0.25 kcal mol^−1^ less accurate than OrbNet Denali.

**Table 1:** Performance comparison on the TorsionNet500 benchmark set. The reference energies are recalculated at their corresponding levels of theory. Metrics evaluated include the percentage of the torsion profiles for which the Pearson correlation coefficient (*R*) is greater than 0.9, the average Pearson *R* over the torsion profiles, the MAE and RMSE of the relative energies of the torsion profiles, and minima accuracy, which is defined as the percentage of torsion profiles where the global minimum of the profile is correct to within 20° and 1 kcal mol^−1^

Method	Pearson *R* > 0.9 (% profiles)	Average profile Pearson *R*	MAE (kcal mol^−1^)	RMSE (kcal mol^−1^)	Minima accuracy (%)
AIMNet2	96.6	0.99	0.32	0.47	98.2
OrbNet denali	99.4	0.99	0.12	0.18	100.0
GFN2-xTB	76.4	0.88	0.73	1.00	94.0
B97-3c	97.4	0.99	0.29	0.43	100.0
ANI-2x	73.2	0.90	1.30	1.90	91.8

### Efficient optimization of molecules to macrostructures

**Fig. 6:**
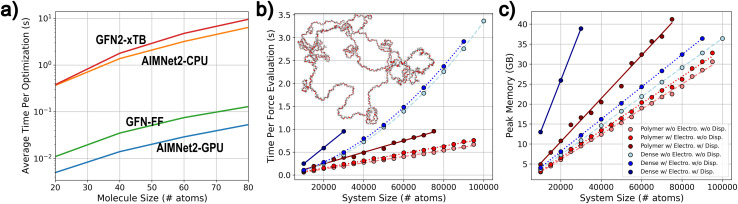
Benchmarking molecular and macrostructure optimization performance of the AIMNet2 architecture. (a) Small molecule optimization performance, defined as the total average time to reach convergence, comparison for GFN2-xTB (red), GFN-FF (green), AIMNet2 using CPU (orange) and GPU (blue) resources. CPU optimizations were performed on a single core of an i7-9700K system, and GPU optimizations on an NVIDIA L40S. (b) Macrostructure time (b) and peak memory (c) for force evaluations. Model systems are random polymer coils (red) and condensed phase methane (blue), where light colors (dashed lines) are for short-range models, dark colors (solid lines) are for models with long-range Coulomb + D3 dispersion, and standard colors (dotted) are for models with long-range Coulomb.

An attractive feature of broadly transferable MLIPs is their ability to enable fast and accurate optimization of an enormous number of molecular and material structures. To highlight this performance for the AIMNet2 architecture, we conducted geometry optimization of varying system sizes, measured computational efficiency and scalability metrics, and compared them with regularly used low-cost methods: GFN-FF and the semi-empirical GFN2-xTB. The efficiency of the AIMNet2 architecture for optimizing small molecule conformer ensembles, *i.e.*, batches of same sized molecules with different initial geometries, is shown in Fig. 6a. GFN-FF, GFN2-xTB, and AIMNet2 (CPU and GPU implementations) exhibit optimization efficiency, defined as the total time to reach convergence, that scales as O(*N*^2^), where *N* is the number of total atoms in the conformer structures. The performance of our GPU PyTorch implementation is particularly notable, where the AIMNet2 architecture yields ∼5× faster optimization in comparison to GFN-FF for systems consisting of up to 80 atoms. This supports an ability to drastically accelerate high-throughput optimization tasks and opens avenues to readily scale to millions of structures with modest resources. Carrying out AIMNet2 geometry optimization on a CPU results in a slower time-to-converge by approximately 2 orders of magnitude, being slightly faster than GFN2-xTB.

It is worth commenting that direct benchmarking between the semi-empirical methods and AIMNet2 is challenging due to the underlying details of the optimizer implementations. Our AIMNet2 small molecule conformer ensemble benchmark uses an in-house batched PyTorch implementation of the FIRE optimizer, which we found to require ∼1.5–2.0× more steps to converge than the approximate normal coordinate rational function optimizer (ANCopt) implemented within the xTB software suite. Despite requiring more gradient calls, we still observe improved performance for AIMNet on both CPU and GPU. Thus, the 5× speed-up can be viewed as a soft lower bound, and refinement of the optimization strategy can lead to even better performance.

For large structure optimization, we examine two classes of systems in different density regimes consisting of up to 10^5^ atoms: polymer random coils of polyethylene oxide (PEO) and condensed phase methane (0.425 g cm^−3^), see Fig. 6b and c. We emphasize that these large systems are selected as model cases to demonstrate the scalability of optimization efficiency afforded by the AIMNet2 architecture, and validating our pretrained models' ability to simulate polymer systems or condensed phase methane is outside the scope of this report. Efficiency is presented in terms of time per force evaluation to remove ambiguity that may arise from arbitrary differences between the initial geometry and converged structures. Moreover, only the performance of AIMNet2 is reported due to the computational limitations of performing semi-empirical optimization for systems of these sizes. O(*N*) scaling per optimization step is observed for both computational time and required memory for polymer systems for systems up to 10^5^ atoms. A single optimization step requires no more than three quarters of a second on a modern GPU, which is largely enabled by memory and thread efficient operations used in constructing the AIMNet2 architecture. For the periodic methane models, the time required for force evaluations scale quadratically with the systems size, which is a consequence of the neighbor list construction as opposed to the AIMNet2 inference (scales linearly). As much as 65% of the inference time is spent on neighbor list operations. For example, carrying out a force evaluation on 9 × 10^4^ atoms methane simulation cell with a model using both short range (5 Å) and long-range (15 Å) components, requires 2.16 s to build the neighbor list but only 0.75 s for AIMNet2 evaluation. To reduce this disparity, high-performing GPU kernels for efficient construction of AIMNet2 neighbor lists is an ongoing research effort. In Fig. 6b and c optimization performance is also reported as a function of long-range interaction types. While the inclusion of Coulomb interactions requires little additional computational effort (both scaling and memory), our pytorch D3 dispersion model is found to produce a significant memory footprint. This presents yet another opportunity for optimized kernel development, which conceivably benefits any MLIP developer seeking to include *post hoc* D3 corrections. It is worth noting that we used a limited memory Broyden–Fletcher–Goldfarb–Shanno (LBFGS) optimizer to measure macrostructure optimization performance using a custom AIMNet2 calculator plugin to the Atomic Simulation Environment (ASE) software, which imposes a small 5% overhead on top of the MLIP energy and force inference. Regardless, the overall efficiency afforded by the AIMNet2 architecture combined with the robust accuracy of our provided pretrained models can be leveraged for high-throughput, chemically diverse, scalable geometry optimization.

### Molecular dynamics

**Fig. 7:**
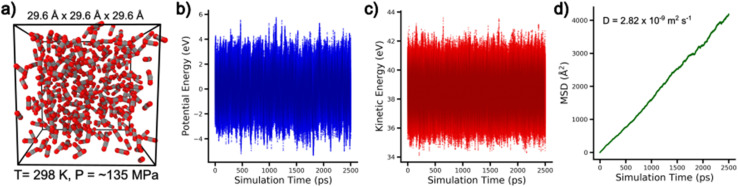
Demonstration of stable molecular dynamics simulations performed with AIMNet2. (a) molecular dynamics snapshot of condensed phase CO_2_ at 298 K. (b) and (c) traces of the AIMNet2 calculated potential energy and the systems kinetic energy over the molecular dynamics simulation, respectively. The potential energy is shifted by the mean values calculated over the last half of the production run to allow for the magnitude of fluctuations to be easily observed. (d) Average mean squared displacement (MSD) of the 1000 CO_2_ over time used to calculate the self-diffusion coefficient.

While the pretrained models provide widespread chemical space coverage, efficient inference, and accurate explicit treatment of nonbonded interactions, it is worthwhile to examine the AIMNet2 architecture's capability for performing molecular dynamics simulations. A recent report by Fu *et al.*^48^ remarked that standard energy and force error metrics used by MLIP model builders are not necessarily reflective of an ability to perform stable molecular dynamics simulations. Explicitly demonstrating such a capability, particularly without equivariant message passing, provides important validation for potential use cases of the AIMNet2 architecture. With this motivation, we assessed the behavior of condensed phase carbon dioxide at 298 K with molecular dynamics simulations, see Fig. 7a. Our decision to examine this model system is twofold: (1) simulating CO_2_ in a dense fluid state with periodic boundary conditions is a clear extrapolatory task as AIMNet2 was trained on small-to-moderately sized gas phase systems and (2) the work of Mathur *et al.*^49^ provides precedent for the expected level of accuracy that CO_2_-specific MLIPs (in their case Deep Potential models^50^) can obtain. It should be noted that the AIMNet2 training dataset does not contain exhaustive sampling of CO_2_ molecule clusters. Therefore, performing stable and reasonably accurate molecular dynamics simulation of the CO_2_ model system serves as an additional measure of the AIMNet2 architecture's generalizability. A complete description of simulation specific details is provided in the Methods section. In addition to demonstrating stability, we calculated the average self-diffusion coefficient by tracking the mean-squared displacement (MSD) over the simulation trajectory and applying the Einstein approach.^51^

In Fig. 7b and c, the potential and kinetic energy throughout the 2.5 ns simulation with data collected every 10 fs are shown. The traces of these energy functions are absent of any aberrations and display fluctuations with magnitudes typical of molecular dynamics simulations performed with classical empirical potentials, indicting no signs of instability. Moreover, we applied molecular geometry-based postprocessing criteria to confirm that all CO_2_ molecules stayed intact and maintain approximately linear geometry, *i.e.*, we did not find any so-called “exploding molecules” that are typical of unstable simulations. In Fig. 7d, we report the calculated MSD, averaging over all 1000 CO_2_ molecules, which exhibits the expected linear relationship in the long-time scale. This results in a self-diffusion coefficient of 2.82 × 10^−9^ m^2^ s^−1^ where the approximate experimental value, interpolated from the work of Groß *et al.*, is 7.09 × 10^−9^ m^2^ s^−1^.^52^ Depending on the DFT functional used for generating reference data and the temperature evaluated, the DeePMD models of Mathur *et al.*^49^ displayed similar disagreement factors of up to 2.5× (also as underpredictions) with respect to the experimental measurements. The error in the AIMNet2 derived self-diffusion coefficient originates from the underlying DFT functional, the model architecture, and the chemical information available in the training dataset. A significant DFT functional dependence for CO_2_ fluid properties has been previously discussed by Goel *et al.*,^53^ which is also observed by Mathur *et al.*^49^ Deconvoluting the degree to which each of these factors contributes to the prediction accuracy is a topic for future study. Regardless, our observations that our MLIP architecture can achieve relatively long timescales (for MLIPs) without noticeable aberrations and display accuracy comparable to previous work, despite any system-specific training, suggesting that AIMNet2 can effectively drive stable molecular dynamics simulations. It is worth commenting that the development of ML potentials to accurately capture a wide range of non-local intermolecular interactions and related properties in a condensed phase system is a non-trivial task,^23^ and the robustness of pretrained AIMNet2 models trained on gas phase calculations translating to condensed phase simulations is under ongoing investigation. For example, the GEMS model of Unke *et al.*,^54^ which uses a divide-and-conquer strategy of training on DFT calculations of molecular fragments, supports the viability of gas phase-to-larger scale MLIP-driven simulations. They describe the necessity to include sizeable molecular systems to accurately learn long-range interaction behavior in heterogenous systems, which is particularly relevant to large-scale molecular simulations such as those aimed at studying protein dynamics. Examples of large noncovalent complexes compose only a small fraction of the AIMNet2 training set in the interest of training efficiency. Consequently, we have demonstrated molecular simulations for homogenous condensed phase CO_2_, but such performance is unlikely to extend to biomacromolecules for example. We emphasize this is a deficiency that is inherited from the aim of the training set, and the AIMNet2 architecture can drive such heterogenous simulations given a sufficient set of targeted training data. In general, the AIMNet2 architecture provides a reliable and efficient method for training models capable of dense periodic boundary simulations; however, we stress that users of our pretrained models should not expect that the molecular and intermolecular complex training set used in this work will yield accuracy in condensed phases. The complete set of scripts needed to train AIMNet2 models can be found in the Code availability section.

### AIMNet2 in the landscape of MLIPs

Reflective of the evolving molecular modeling capabilities enabled by MLIPs, the introduction of new models with diverse use cases has grown in recent years. From a high-level perspective, these interatomic potentials can be classified according to model balance and model objective, which are the main influencers dictating algorithmic design and training dataset construction. In this section, we aim to formally state the balance and objective targets of our pretrained models and provide an overview of the AIMNet2 architecture's capabilities in comparison to other modern MLIPs. Model balance can be regarded as management of MLIP accuracy, efficiency, and transferability, which are defined by intertwined relationships that are akin to the performance trade-offs found in traditional molecular simulations, albeit on a different scale. We refer to model objective as the models intended use, which is crucial to interpret in the context of the trade-offs described by model balance.

By our assessment, many modern MLIP models tend to favor improvements in accuracy over computational efficiency, *e.g.*, NEQUIP,^55^ Allegro,^56^ TensorNet,^57^ or MACE.^58^ That is not to say efficiency is not a focus of these models. For example, Allegro is a creative solution to offer better computational efficiency than NEQUIP with only modest differences in accuracy. Instead, we emphasize that these architectures have an overall greater computational expense. A recent demonstration from Gao *et al.*^59^ emphasizes this point, where DP-MP models, a message passing variant of the Deep Potential architecture, show ∼2 orders of magnitude faster inference than those equivariant models listed above at the cost of ∼10 meV Å^−1^ force accuracy. AIMNet2 achieves similar computational performance, depending on the use of sparse or dense operations and neighbor list construction, while being slightly less accurate than MACE or NEQUIP when more computationally demanding yet informative higher body-order terms are included. The optimization of the SNAP potential by Wood and Thompson is another example.^60^ Although this was reported prior to the models mentioned above, their thorough discussion about the performance of MLIPs for pragmatic molecular simulations maintains its relevance.

**Fig. 8:**
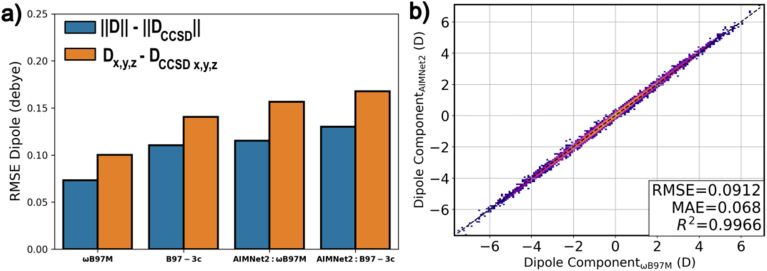
Performance of pretrained AIMNet2 models for dipole inference. (a) QM7b couple-cluster (CCSD) benchmark for dipole norm (blue) and dipole components (orange). (b) Parity between the AIMNet2 model trained to ωB97M data and the same level of reference DFT on QM7b structures.

The objective of the pretrained AIMNet2 models is to provide reliable accuracy for general molecular modeling at an affordable computational cost, ultimately meeting the varied needs of high-throughput computational chemistry. Other MLIP models have prioritized stable and/or scalable molecular simulations as a main objective, for instance, sGDML,^61^ SNAP,^62^ or DP.^63^ sGDML is particularly interesting for performing molecular simulations because of its scalability and inherent smoothness. However, kernel methods typically suffer from poor transferability, and, as a result, it remains unclear if the sGDML approach can be used for general chemistry without system-specific or domain-specific retraining, which is a key benefit of AIMNet2. It is worth reiterating that the comparisons presented in this section can only be stated for neutral systems, while AIMNet2 takes a step further and includes explicit treatment of molecular charge. Conceivably, the 4GNNP architecture of Ko *et al.*^21^ could be used to train a comparable general MLIP model; however, to achieve this for 14 elements would be a demanding task considering the poor scaling of Behler–Parinello symmetry functions and charge equilibration (QEq) scheme. An extension of DP models capable of predicting Wannier centroid positions for charged compounds is also possible;^22^ however, the algorithmic imposition of net charge would need to be developed and a dataset rivaling AIMNet2 is not available. AIMNet2 occupies a unique space in the MLIP landscape, being broadly applicable across compounds containing common non-metals/halogens, regardless of charge state, while maintaining a high-level of computational efficiency, scalability, and practical accuracy. As an example, the dissociation of charged complexes is one limiting case. This is a consequence of charge redistribution *via* NQE occurring as a function of learned short-range descriptors. As non-neutral species move beyond the message passing cutoff, without intermediary molecules, the lack of communication between system components can yield systematic misprediction. This is an inherent limitation of any MLIP potential that relies on short-range learned descriptors, such as those used in message passing. Regardless, for molecules and molecular complexes, the pretrained AIMNet2 models are observed to produce distributions of atom-centered point charges with accuracy near that of DFT. In Fig. 8a, a comparison of predicted dipoles with respect to coupled cluster calculations for AIMNet2 (calculated from the distribution of point charges) and reference DFT (using the electron density) is presented for the QM7b dataset.^64^ AIMNet2 models trained to ωB97M-D3 data are found to be ∼0.04 D less accurate than the same underlying DFT and provide a similar quality of predictions as B97-3c. A direct comparison between the AIMNet2 and ωB97M dipole components is provided In Fig. 8b, which shows strong correlation, *R*^2^ = 0.99, and modest RMSE, 0.09 D. Similar results are found for the dipoles AlphaML dataset,^64^ see the parity plot in Fig. S6,† which contains slightly larger organic molecules than QM7b.

## Conclusion

Simulation methods and molecular modeling tasks using MLIPs are becoming increasingly mature and will, likely, continue their growth as emergent core components in computational chemistry research. By our assessment, the field of MLIP development is beginning to split into several distinct focus areas, such as being exceptionally accurate for specific systems or being efficient and broadly generalizable with practical accuracy for many applications. Regarding the first area, advances are mainly being achieved by the development of increasingly complex and/or expressive model architectures, for example, the recent embrace of equivariant models.^55,65,66^ For the second area, which is the primary focus of our pretrained AIMNet2 models, we show that systematically curating an expansive dataset, allowing our model to learn its own flexible representations, and including physics-based functional forms into the MLIP architecture yields significant progress. Notable contributions to the performance of AIMNet2 are the imposition of net charge, an ML-assisted charge redistribution scheme (Neural Charge Equilibration or NQE^25^), and convolutions for partial charge updating, all of which incorporate rich electronic structure information to enhance the learning process. It is worth commenting that work by Ko *et al.*^21^ also experienced significant gains in performance by including electronic structure information, albeit using a different strategy of predicting partial charges with an ML-parameterized charge equilibration technique that served as inputs alongside Behler–Parinello symmetry functions. In contrast to QEq, the NQE scheme scales linearly and introduces negligible computational overhead.

In this work, we report an improved atoms-in-molecules neural network potential, AIMNet2, which yielded a set of pretrained models for diverse organic and elemental-organic compounds. The AIMNet2 architecture overcomes many of the limitations intrinsic to the original model. In particular, AIMNet2 explicitly includes long-range interactions so that it is not bound by the locality of message passing, it is applicable to neutral and charged states, and covers compounds composed of twice as many (14) different chemical elements. Although it was not highlighted in this report, the multi-task predictions of the 1^st^ AIMNet model can easily be incorporated into AIMNet2 by, for example, including additional predictive neural networks that operate on the learned AIM representation. The result is a flexible MLIP model that can be readily tailored to predict additional chemical properties without having to retrain the entire model for each task.

As a final note, it is worth commenting on the challenge of achieving full chemical space coverage. Setting aside issues with the transferability of the underlying reference data, it remains uncertain what is required, or if it is even possible or necessary, to train a single universal neural network potential with sufficient accuracy and efficiency for any task. Considering the surprising, at least in our opinion, generalizability of AIMNet2, it is clear that including information derived from electronic structure and interfacing with known physics-based functional forms are crucial steps in the right direction. While there are some physical phenomena that still need to be addressed, *e.g.*, reactions or open-shelled species, our validation checks, benchmarking, and efficiency tests support the idea that AIMNet2 is a suitable drop-in replacement for DFT in many computational chemistry practices without needing to be retrained.

## Methods

### Dataset preparation

To create the overall pool of training data we selected neutral and charged molecules under 20 heavy atoms from PubChem^34^ and ChEMBL^33^ databases that contained species in our defined set of elements {H, B, C, N, O, F, Si, P, S, Cl, As, Se, Br, I}. All realistic tautomeric forms and protonation states across the pH range (1–14) were generated with Chemaxon JChem software.^67^ We utilized geometry optimization, torsional profile scans, and molecular dynamics (MD) as primary methods to explore molecular PESs around their minima. Thermal fluctuations of atoms in MD simulations allow for the near-equilibrium sampling of molecular conformational space. MD simulations of small molecular clusters were used for expanded sampling of noncovalent interactions. The set of structures was supplemented with systems from ANI-1x,^17^ ANI-2x^18^ and OrbNet^36^ datasets to provide broader chemical space coverage in the AIMNet2 training set. Additional details, such as dataset statistics, are provided in the SI. 3Similar to our previous work,^25^ we used quantum mechanically derived force field (QMDFF)^68^ as an efficient method to construct system-specific and charge-specific potential for a molecule. We also applied the GFN2-xTB^35^ tight-binding model to obtain relaxed conformations, force constants, charges, and bond orders that are needed for the QMDFF model.

Molecular clusters were created by constructing a rectangular periodic cell within the range of 20 to 30 Å. *N* = 2–5 molecules from dataset are then selected randomly, with a probability that is skewed toward choosing molecules with less non-hydrogen atoms. The selected molecules are then embedded within the periodic cell with random positions and orientations under the condition that no two atoms in different molecules are within 1.5 Å. The atom density of the box is also randomly determined within reasonable bounds. Preliminary AIMNet2 models are used to run an MD simulation on the constructed box of molecules. MD is carried out at a random temperature between 50 K and 600 K using the Langevin thermostat. After 100 timesteps, the box is decomposed into a complete set of *N*-mer structures {*x*_*i*_}, where *i* indexes the molecules. Only *N*-mer structures with at least two atoms, one from each monomer, within a distance cutoff of 6.0 Å are selected.

For torsion sampling component of the AIMNet2 dataset construction, SMILES strings are selected from a subset of molecules with rotatable dihedrals. Consistent with the diversity selection algorithm (see below), we selected all possible conformers with unique torsion angles. RDKit is used to embed the molecules in 3D space and select rotatable dihedrals.^69^ The preliminary AIMNet2 models are used to optimize the starting geometry, and carry out a relaxed scan, incremented by 10° over the entire torsion profile. All DFT calculations were performed with the ORCA 5 (ref. 70) package using B97-3c^32^ and ωB97M-D3/def2-TZVPP^37^ levels of theory.

### Model training

AIMNet2 models were trained using minibatch gradient descent with the AdamW^71^ optimizer. To improve training performance, all minibatches were composed of molecules with the same number of atoms to avoid padding operations. Proper data feed shuffling was achieved within the multi-GPU distributed data-parallel (DDP) approach: gradients on model weights were averaged after 8 random batches were evaluated in parallel, thus the effective combined batch size was 2048. Training was performed on 8 Nvidia V100 GPUs. We employ a reduce-on-plateau learning rate schedule, which leads to training convergence within 400–500 epochs. The training objective was minimization of weighted multi-target mean squared error (MSE) loss function:
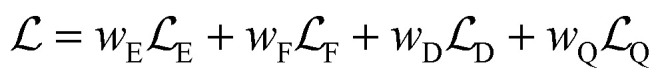


The loss functions include the weighted contributions from total energy prediction error 
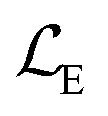
 (scaled by the square root of number of atoms within molecule), partial charges prediction error 
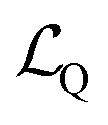
, and errors of prediction of the components of atomic forces 
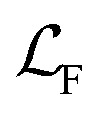
, total dipole 
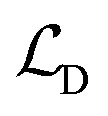
 and total quadrupole 
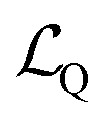
. The sum of the weights was normalized to unity, where values of *w* were selected *via* an empirically guided hyperparameter search. The final AIMNet2 loss contribution weights were 1.0, 0.2, 0.05, and 0.02 for *w*_E_, *w*_F_, *w*_D_, and *w*_Q_, respectively using units based on eV, Å, and electron charge. The partial charges inferred by AIMNet2 are learned such that they reproduce the molecular dipole and quadrupoles extracted from the DFT reference calculations.

### Data distillation

The main purpose of the AIMNet2 model is to predict the energy, atomic forces, and charge distribution of organic and element-organic molecules in equilibrium and non-equilibrium configurations. The amount of required data could be drastically reduced with active learning techniques, such as the selection of the most important samples (molecular configurations) to label (compute reference DFT properties) and include in the training dataset. For example, the original 2.0 × 10^7^ ANI-1 dataset for neutral CHNO organic molecules was reduced to 4.5 × 10^6^ active learning. Extension to just three extra chemical elements S, F and Cl required additional 4 × 10^6^ samples. Therefore, a comparable extension of that dataset to 7 extra elements (B, Si, P, Br, As, Se, I), and charged molecules could be expected to require an order of ∼10^8^ new DFT data points, which is approaching practical limits. Therefore, to reduce the dataset even further, we combined our standard active learning query-by-committee approach^14,15,72^ with data distillation.^73,74^

The process of data distillation involves two main components: a teacher (T) dataset and student (S) training. The teacher dataset is composed of all available labeled data. One could train an MLIP to the full teacher set to achieve a potential that captures the underlying physical and chemical relationships defined in the data. However, labeling the full teacher dataset with higher level of theory DFT calculations is impractical, even with supercomputing resources, and therefore, data distillation can be applied to limit redundant chemical information such that a tractably sized training set can be obtained. If D represents a general dataset, *f*_*θ*_ represents an MLIP model with parameters *θ*, and *f*_*θ*_(*x*) is the model's prediction for data point *x*, then the expected loss for dataset D in relation to *θ* is
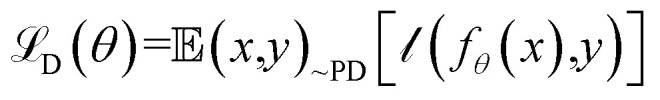
where *x* and *y* are the input data and label pair from D, 
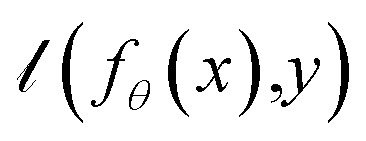
 is the given loss value between the prediction and ground truth. Dataset distillation aims to reduce the size of large-scale training input and label pairs T = {(*x*_*i*_, *y*_*i*_)} by creating smaller student pairs S = {(*x*_*i*_, *y*_*i*_)}, so that models trained on both T and S can achieve similar performance, which can be formulated as:
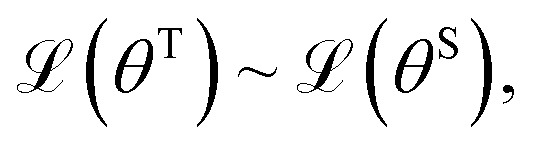
where *θ*^T^ and *θ*^S^ are the parameters of the models trained on S and T respectively. In our case, we focus on so-called distilling in instead of distilling out. In the distilling process, the student dataset is built up iteratively as a subset of the teacher (master) dataset.

### Diversity selection

Molecular species used in our benchmark Section were collected *via* diversity selection using the local environment of each non-hydrogen atom composing the CSD-extracted molecules. Specifically, for each atom, we utilized a hashing function operating on atomic number, number of connected hydrogen atoms, the total number of neighbors, and the same set of properties for all neighboring atoms. This hash uniquely encodes the local environment for each atom in a molecule, and comparing hash values was our strategy for discerning molecules with diverse chemical structures. For each of the 14 atomic species types covered by the pretrained AIMNet2 models, we selected 10 molecules that contain the least frequent atomic hashes. Some of these top-10 molecules were duplicated. As a result, the final number of benchmark structures was reduced to 113 molecules instead of 140 after enforcing uniqueness. These 113 molecules exemplify a selection of the most unusual chemical bonding present in CSD, and thus serve as challenging test cases for demonstrating MLIP applicability. The full list of molecules and reference codes are supplied in the ESI.†

### MD simulations

The molecular dynamics simulation for the condensed phase CO_2_ system was performed using the atomic simulation environment (ASE)^75^ with a custom calculator (see the AIMNet2 repository). The simulation was performed under constant number of particles, volume, and temperature (NVT) conditions *via* the application of a stochastic velocity rescaling thermostat developed by Bussi, Donadio, and Parrinello.^76^ This thermostat has been verified to correctly sample the canonical ensemble, provides proper conserved quantities, and produces accurate self-diffusion coefficients in fluid phase water. The NVT simulations were carried out with a reference temperature of 298 K, 0.5 fs timestep, and a characteristic thermostat time constant of 100 fs. Initial velocities were assigned by sampling a Maxwell–Boltzmann distribution at 298 K, which were then adjusted to set the total translation and rotational momenta of the system to zero. We verified that these net momenta were conserved during postprocessing of the simulation results. Long-range dispersion and electrostatic interactions were applied using a neighbor list built over a 15 Å cutoff at every timestep. We elected to account for electrostatic interactions using the damped shifted force method,^77^ which our initial testing showed to be a suitable choice for the CO_2_ system to achieve computationally efficient (O(*N*)) scaling without incurring differences to the dynamics compared to common long-range solvers, for example, Ewald summation.^78^ The initial system was prepared using the enhanced Monte Carlo (EMC) software developed by In't Veld and Rutledge,^79^ where 1000 CO_2_ molecules were packed into a simulation cell at a density of ∼0.95 g cm^−3^ and relaxed using an empirical potential. Prior to molecular dynamics, an LBFGS minimization for 10^3^ steps and a max displacement of 0.02 Å per step was performed using the AIMNet2 pretrained model to limit any unfavorable initial geometries that may result from differences between the empirical potential and our MLIP. To compare diffusion coefficients, the external pressure was calculated using the equations described by Thompson, Plimpton, and Mattson,^80^ which was then matched to the corresponding state point (∼135 MPa and 298 K) through simple interpolation of the experimental results.

## Code availability

The trained AIMNet2 models, training scripts, and the code to reproduce this study is available in GitHub at https://github.com/isayevlab/aimnetcentral.

## Author contributions

D. M. A., R. Z., and O. I. designed the study. D. M. A. and R. Z. developed and implemented the method. R. Z. prepared all QM data. D. M. A. and R. Z. ran calculations and created all display items. All authors contributed to the writing of the paper and analysis of the results.

## Conflicts of interest

There are no conflicts to declare.

## Supplementary Material

SC-016-D4SC08572H-s001

## Data Availability

The molecular structures in the training datasets used in this study are publicly available at https://doi.org/10.1184/R1/27629937.v2.
