# Supplementary material for: AIMNet2: a neural network potential to meet your neutral, charged, organic, and elemental-organic needs
Source: Chem Sci. 2025 Apr 29;16(23):10228–44. doi: 10.1039/d4sc08572h (PMC12057637; doi:10.1039/d4sc08572h)
Supplement: SC-016-D4SC08572H-s001 [file SC-016-D4SC08572H-s001.pdf]

Supplementary Information (SI) for Chemical Science.
This journal is © The Royal Society of Chemistry 2025

Supplementary Information

AIMNet2: A Neural Network Potential to Meet your Neutral, Charged, Organic, and Elemental-Organic Needs

Dylan M. Anstine†, Roman Zubatyuk†, Olexandr Isayev*

[1]*Department of Chemistry, Mellon College of Science, Carnegie Mellon University, Pittsburgh, Pennsylvania 15213, USA*

†*Equal contributions*

* *Correspondence: olexandr@olexandrisayev.com*

- **Supplementary Table 1:** Number of molecules and conformers in training and test datasets.
- **Supplementary Figure 1:** Distribution of molecule sizes in training and test datasets.
- **Supplementary Figure 2:** Distribution of elements in training and test datasets.
- **Supplementary Figure 3:** Distribution of molecular charges for training and test datasets.
- **Supplementary Note 1:** Diverse element-organic CSD benchmark set.
- **Supplementary Table 2:** Benchmark performance statistics of GFN2-xTB and two AIMNet2 variants against experimentally observed geometries with diverse element CSD conformation benchmark set.
- **Supplementary Figure 4:** Distribution of RMSD for dihedral angles of GFN2-xTB and two AIMNet2 variants against experimentally observed geometries in diverse element CSD conformation benchmark set.
- **Supplementary Note 2:** CSD conformer benchmark set
- **Supplementary Tabe 3:** Benchmark performance of various methods on CSD conformer benchmark set
- **Supplementary Figure 5:** Distribution of RMSE and MAE errors for various
- **Supplementary Table 4:** MAE for energy predictions (kcal mol^{-1}) on GMTKN55 subsets

Supplementary Table 1: Number of molecules and conformers in training and test datasets.

Source	# of samples	% of dataset
ANI-1x	635626	3.1
ANI-2x	365980	1.8
Orbnet	505216	2.5
Peptide dimers	39618	0.2
ChEMBL molecules	1313538	6.5
PubChem MD	6779541	33.3
PubChem opt	3107211	15.3
PubChem torsion scans	2647778	13.0
PubChem clusters MD	2299495	11.3
PubChem openbabel	1194665	5.9
PubChem Omega pose	1439915	7.1
Total:	20328583	

Supplementary Figure 1: Distribution of molecule sizes in training and test datasets.

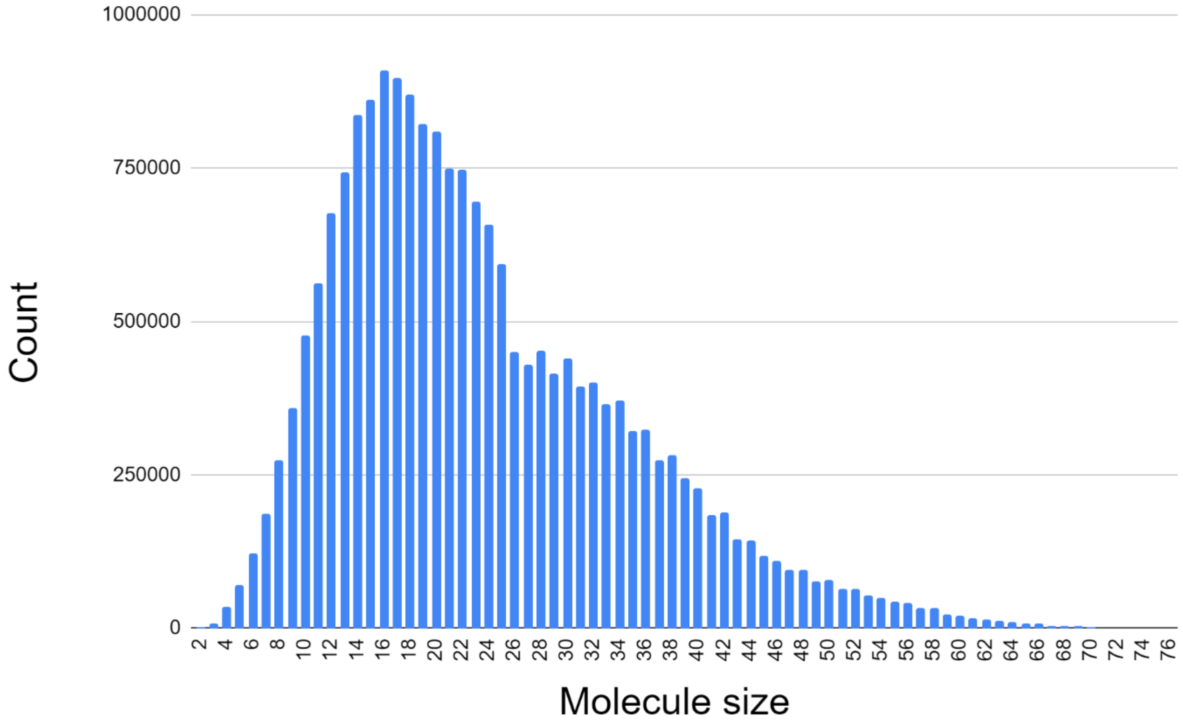

Supplementary Figure 2: Distribution of elements in training and test datasets.

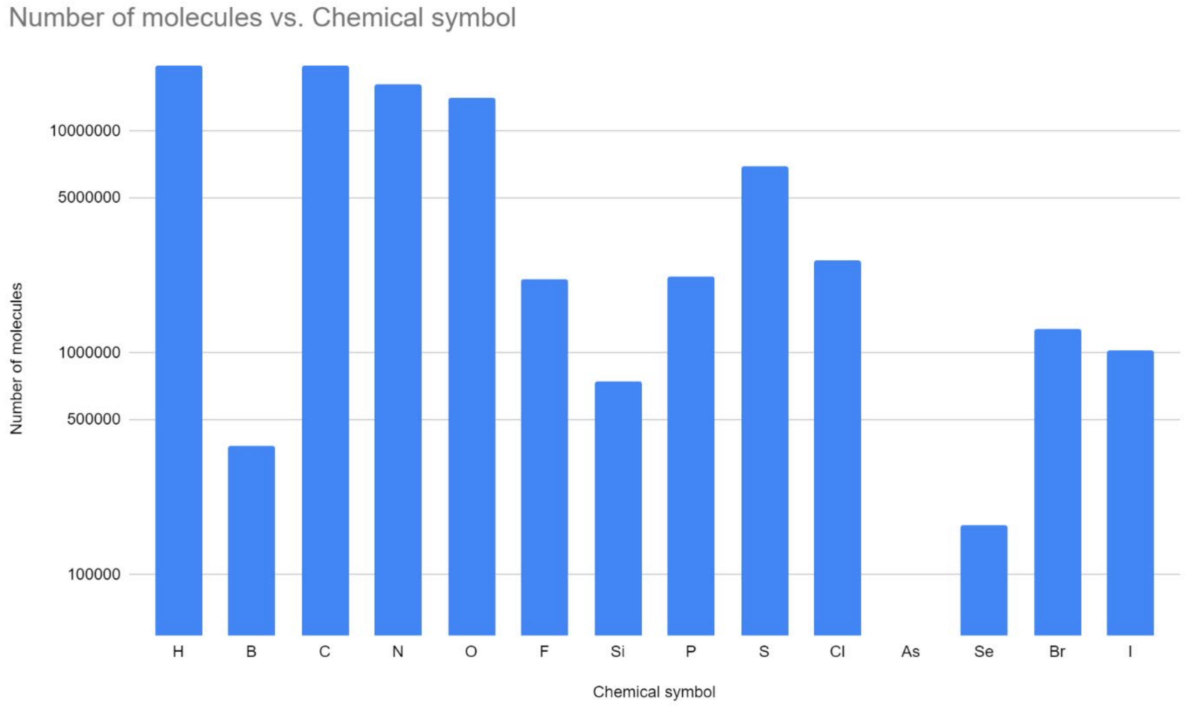

Chemical symbol	Number of molecules	% of molecules
H	19781827	98.38
B	382762	1.90
C	19693001	97.93
N	16313383	81.13
O	14084153	70.04
F	2135500	10.62
Si	747543	3.72
P	2197353	10.93
S	6963661	34.63
Cl	2601176	12.94
As	53125	0.26
Se	167645	0.83
Br	1284649	6.39
I	1027304	5.11

Supplementary Figure 3: Distribution of molecular charges for training and test datasets.

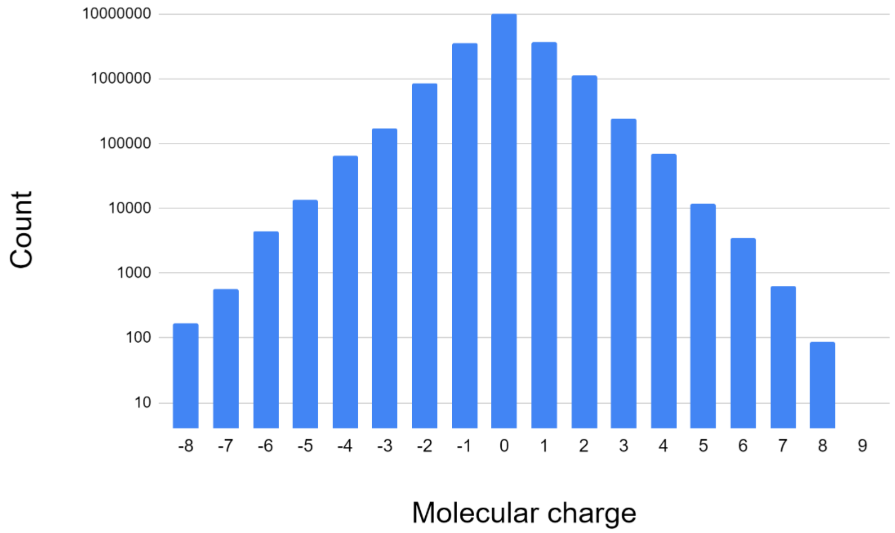

Charge	Count
-8	168
-7	565
-6	4490
-5	13486
-4	65374
-3	174089
-2	846841
-1	3580002
0	10230127
1	3737784
2	1129289
3	242165
4	68381
5	11598
6	3474
7	641
8	87
9	4

Supplementary Note 1: Diverse element-organic CSD benchmark set

To select molecular species for our benchmark, we applied diversity selection using the local environment of each non-hydrogen atom composing the CSD extracted molecules. Specifically, for each atom, we applied a hashing consisting of atomic number, number of connected hydrogen atoms, and total number of neighbors, as well as the same set of properties for all neighboring atoms. This hash uniquely encodes the local environment for each atom in a molecule, and, by comparing hashes, molecules with diverse chemical structures can be discerned. For each of the 14 atomic species covered by AIMNet2, we selected 10 molecules which contain least frequent atomic hashes. However, some of these top-10 molecules were duplicates, and the final number of benchmark structures was reduced to 113 molecules instead of 140 after enforcing uniqueness. These 113 molecules exemplify a selection of the most unusual chemical bonding present in CSD, and thus, serve as challenging test cases for demonstrating machine learned potential applicability.

Refcode List:

AGIPAI
AHUDEO
AHULUM
AXEPOI
BAQKAF
BUCXIJ
CIXYAM
DAQGEH_02
DEQXOP
DIDJAE
DISDOB
EZIVOZ
FOXNEL10
GEHVIY10
GONFUM
HAJJAD
HEQZEL
HIJHEQ
HIYWUH
HIYXIW
HOGWIK
HOJMEY
HOWTOC
HUBXOR
HUBXUX
HUGMAX
HULLIM

ICEQIT
ILAXIC
IRABEI
ITIQIM01
IVEBEQ
IZOLUE
JALSOE
JARNAR
JOCCIO
JOFWAC
JONZUH
KEWZOE
KIXLAE
KOXXAZ
LAVGAQ
MAKJEO
MAKKAL
MAXGAT
MOQXUM
NACSEQ
NIMPOO
NOJSUB
NOKQEM
NONCAV
NUBNEF
NUJDEE
OKALET
OKALUJ
OMOHUU
PAJDEJ
POVWUS
POWQID
PUPGIQ
QAFNAN
QIBWON
QOKKIN
QOXWOR
QOYNEA
QUFSIT
QUFSOZ
QUKTAR
RAZROZ
REWXAS

RICLUL
ROZZOY
RUMQEX
SAXREP
SEDMAP
SEDVUV
SIKCIY
SIRNEM
SOQQAR
SUKQIB
TAJHAQ
TEVKUB
UCAVOL
VEPXAQ
VIRDEF
VIYROK
VORWAD
VOZHIE
VOZKOK
VUFRIX
WABNUJ
WEBJUI
WEYYOO
WIHJIG
WOQSEA
WUFZEC
WUKSEA
XIDKOK
XIJSIS
XIQRIB
YARCEA
YESVOH
YESWAU
YETCAB01
YIHRUD
YUNQEG
ZADLOF
ZEXVON
ZOWGEZ

Supplementary Tabe 2. Benchmark performance statistics of GFN2-xTB and two AIMNet2 variants against experimentally observed geometries with diverse element CSD conformation benchmark set

Method	# of broken structures (RMSD > 5 Å)	Mean RMSD, Å	Pearson R for bond lengths	Mean absolute relative deviation in bond length [(d1 - d2) / d1]
GFN2-xTB	2	0.37	0.950	2.4%
AIMNet2-B973c	0	0.32	0.961	2.4%
AIMNet2-wB97M-D3	1	0.35	0.953	2.1%

Supplementary Figure 4: Distribution of RMSD for dihedral angles of GFN2-xTB and two AIMNet2 variants against experimentally observed geometries in diverse element CSD conformation benchmark set.

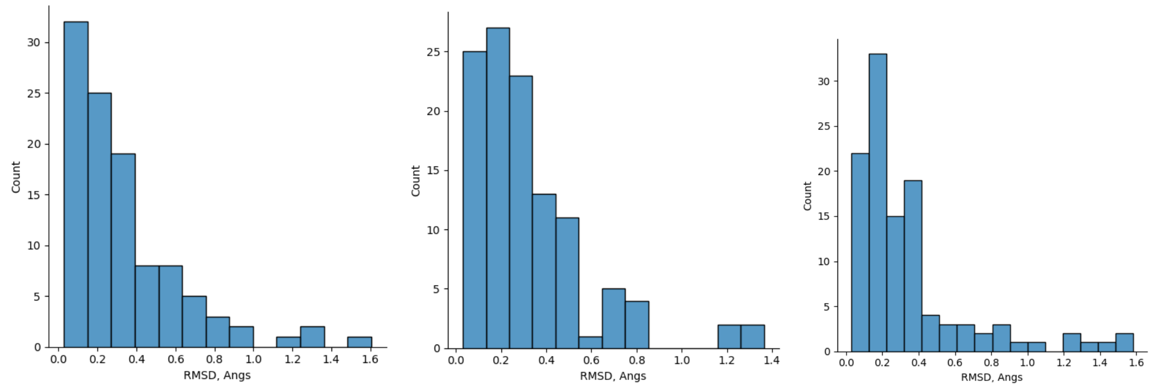

GFN2-xTB AIMNet2-B973c AIMNet2-wB97M-D3

Supplementary Note 2: CSD conformer benchmark set

Ground truth experimental geometries were obtained from the CSD. Beginning with our subset of 250k extracted molecules, we selected a diverse set of 676 molecules which have 10-40 non-H atoms and 1-3 rotatable bonds. From each molecule's SMILES representation an initial pool of molecular structures was produced with OpenEye Omega Dense conformer ensemble generator. On average, 86 distinct conformers were generated for each molecule. After optimization of all conformers within the ensemble, we selected only those within 6 kcal/mol from the lowest energy conformers, which is a typical level of energy cutoff in a conformational search task. Within this pool of molecular geometries, we searched for the conformation which is closest to the experimental structure and recorded its RMSD and relative energy.

It should be noted that this conformational search task also employed diversity selection and, therefore, is intentionally focused on structures with unusual chemistry. The benchmark is, by design, more challenging than a random sampling of molecules from a large database, even though only the molecules for which OpenEye Omega has MMFF94 force field parameters were considered.

As a general comment, there is no guarantee that gas-phase quantum chemical calculations, regardless of their level of theory, would reproduce the molecular conformation observed in a crystal; thus, some irreducible error in geometry predictions and relative energies results from the effects of the crystalline environment. However, in most cases, gas phase calculations can produce conformers which display a relatively close geometry to those that are experimentally resolved from crystals. Therefore, to measure success in this conformational search task we consider the fraction of molecules for which the selected conformer is geometrically close to the experimental structure (RMSD < 0.5A) and has low relative energy in the optimized ensemble ($\Delta E < 2$ kcal/mol). We report statistics for neutral and charged molecules (61% and 39% of the dataset, respectively), which is motivated by crystal field effects generally producing larger deviations in polar environments.

Supplementary Tabe 3. Benchmark performance of various methods on CSD conformer benchmark set

	RMSD < 0.5A			ΔE < 2 kcal/mol			RMSD < 0.5A and ΔE < 2 kcal/mol		
	Neutral	Charged	All	Neutral	Charged	All	Neutral	Charged	All
B97-3c	86.4	78.7	83.4	90.8	81.5	87.2	80.8	66.3	75.2
GFNFF	47.6	55.6	50.7	83.0	82.9	82.9	39.7	45.7	42.1
GFN2-xTB	61.1	41.9	53.6	84.3	79.5	82.5	53.8	31.6	45.2
AIMNet2-B973c	88.9	88.5	88.7	87.8	78.2	84.1	78.9	70.9	75.8
AIMNet2-wB97M-D3	91.4	87.6	89.9	88.6	79.1	84.9	81.4	70.9	77.3

Supplementary Figure 5: Distribution of RMSE and MAE errors (kcal/mol) for various subset of the dataset depending on the total molecular charge

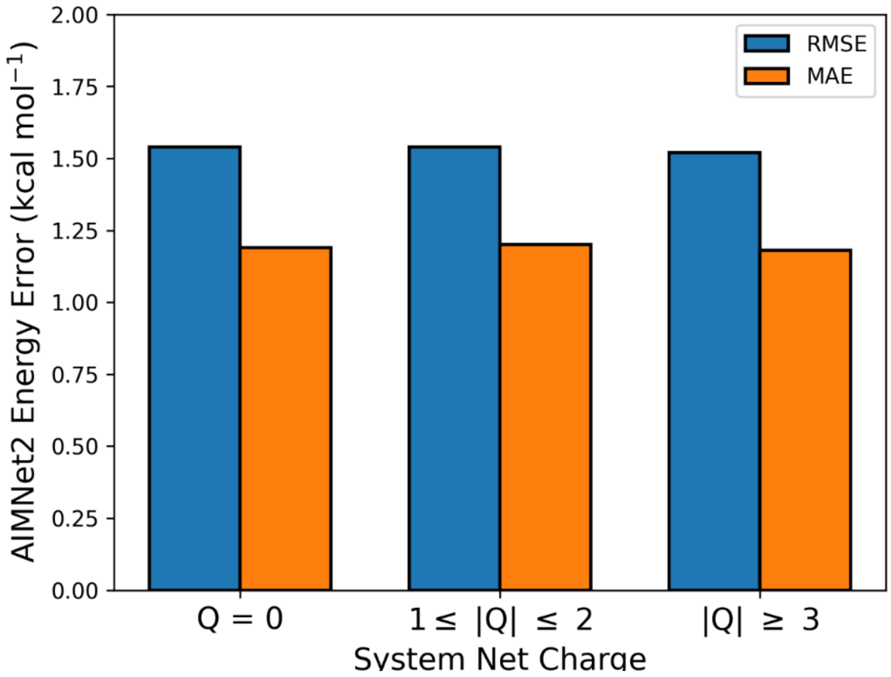

Supplementary Tabe 4. Mean Absolute Energy Errors (kcal mol^{-1}) on GMTKN55 subsets

Subset	wB97x	B97-3c	OrbNet Denali	GFN2-xTB	AIMNet2 B97-3c	AIMNet2 wB97M-D3
PA26	3.32	5.58	5.29	163.05	5.71	3.33
NBPRC	1.67	1.56	10.27	10.51	4.76	1.50
FH51	2.47	4.42	3.58	11.41	4.51	1.49
TAUT15	1.05	1.71	1.13	0.98	1.68	0.74
DARC	1.27	4.34	1.31	17.77	4.53	1.04
BSR36	4.36	1.90	0.77	2.76	9.72	9.10
CDIE20	0.72	1.98	0.61	1.80	1.77	0.69
ISO34	1.18	1.87	1.21	6.90	1.73	0.76
ISOL24	2.75	5.19	2.64	11.68	5.29	2.40
C60ISO	1.18	6.27	11.82	5.80	47.42	17.28
PArel	0.67	1.80	1.60	5.86	2.99	0.94
BHPERI	2.85	4.59	4.72	10.24	2.86	6.61
BHDIV10	1.01	5.80	6.83	8.12	4.86	4.44
INV24	1.63	1.96	4.59	3.32	3.66	2.90
BHROT27	0.47	0.61	0.39	1.17	0.87	0.84
PX13	3.18	7.08	14.84	2.74	5.56	7.46
WCPT18	2.14	5.46	4.91	3.84	6.54	4.28
ADIM6	0.36	0.53	0.40	1.15	2.17	1.48
S22	0.36	0.29	0.45	0.76	0.98	0.89
S66	0.52	0.32	0.48	0.73	0.81	0.63
WATER27	14.23	9.41	2.39	3.05	5.04	8.68
CARBHB12	0.83	2.07	0.91	1.79	0.66	2.39
PNICO23	0.38	1.64	1.71	1.11	2.28	3.42
HAL59	0.34	1.62	2.15	1.28	1.60	2.52
AHB21	3.40	3.27	1.81	2.97	1.71	1.44
IL16	2.09	2.34	4.60	4.32	2.27	1.29
IDISP	2.78	3.91	2.61	6.78	4.59	1.35
ICONF	0.34	0.38	1.25	1.63	0.92	0.66

ACONF	0.09	0.21	0.06	0.19	0.26	0.17
Amino20x4	0.26	0.33	0.35	0.95	0.52	0.54
PCONF21	0.33	0.83	0.47	1.76	1.04	1.01
MCONF	0.48	0.33	0.42	1.72	0.61	0.43
SCONF	0.30	0.77	0.32	1.64	1.44	1.30
UPU23	0.94	0.51	0.87	2.91	1.56	2.02
BUT14DIOL	0.41	0.41	0.40	1.25	0.19	0.15
